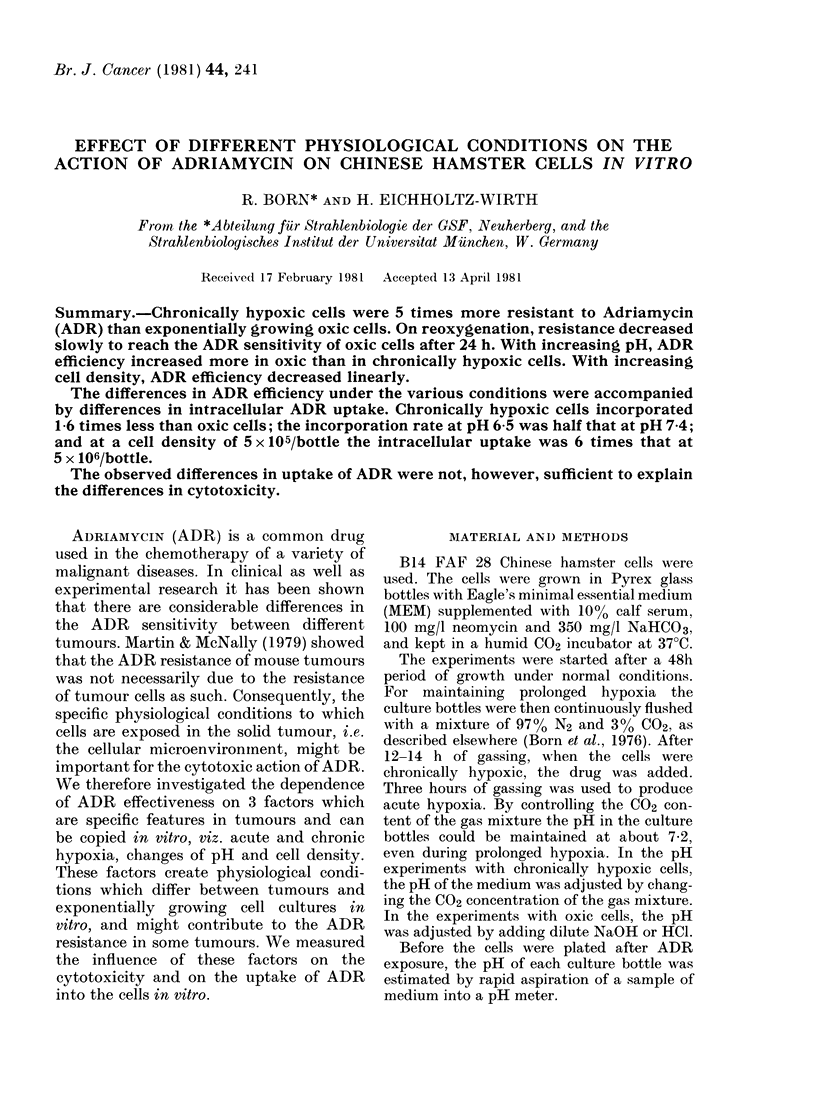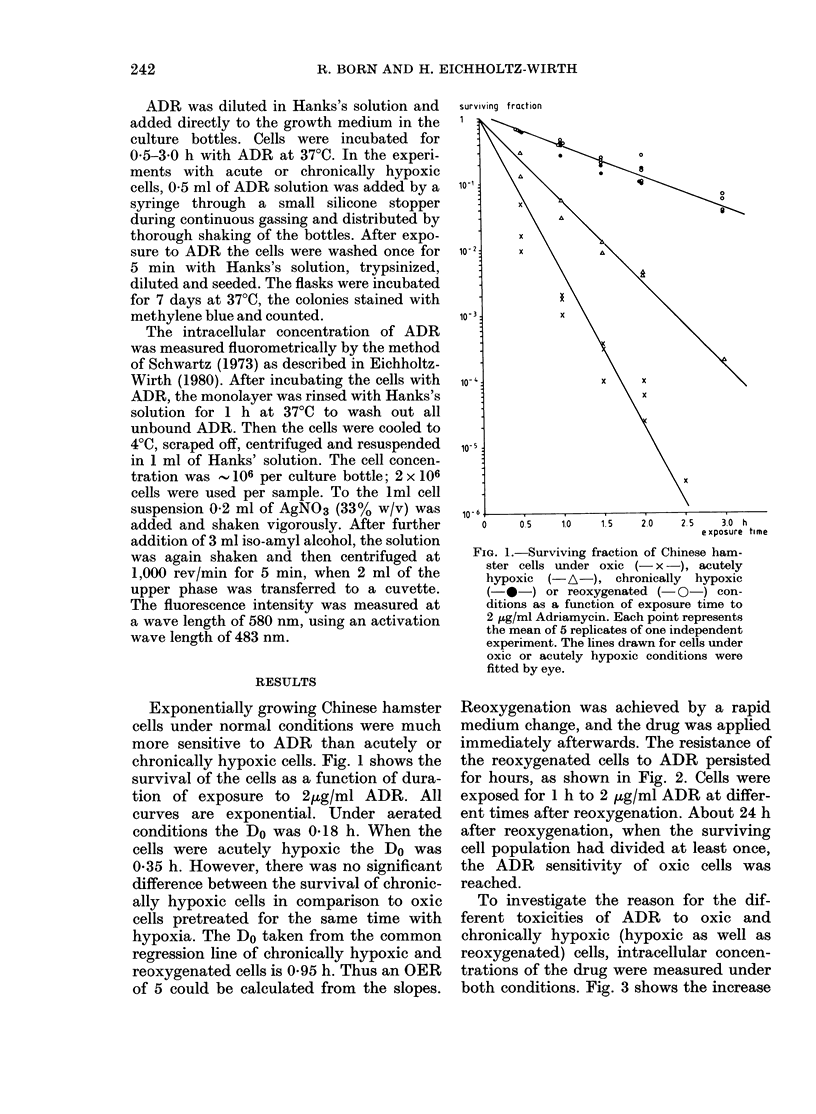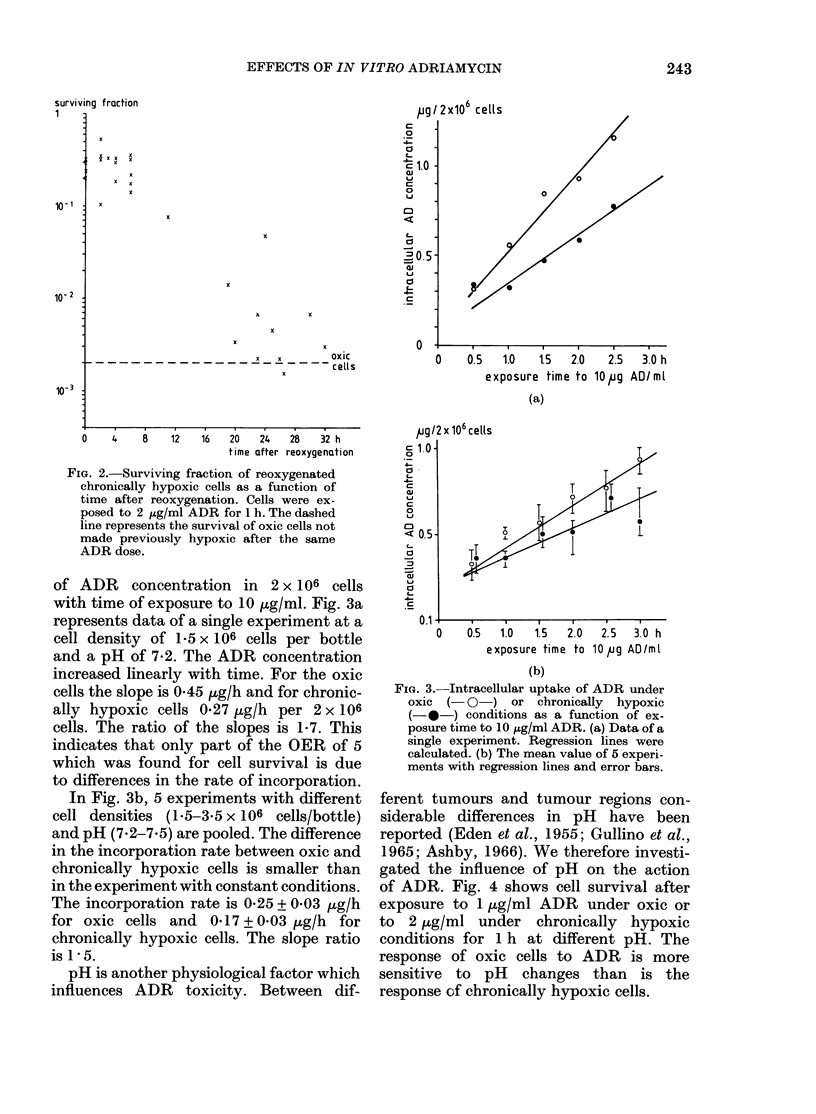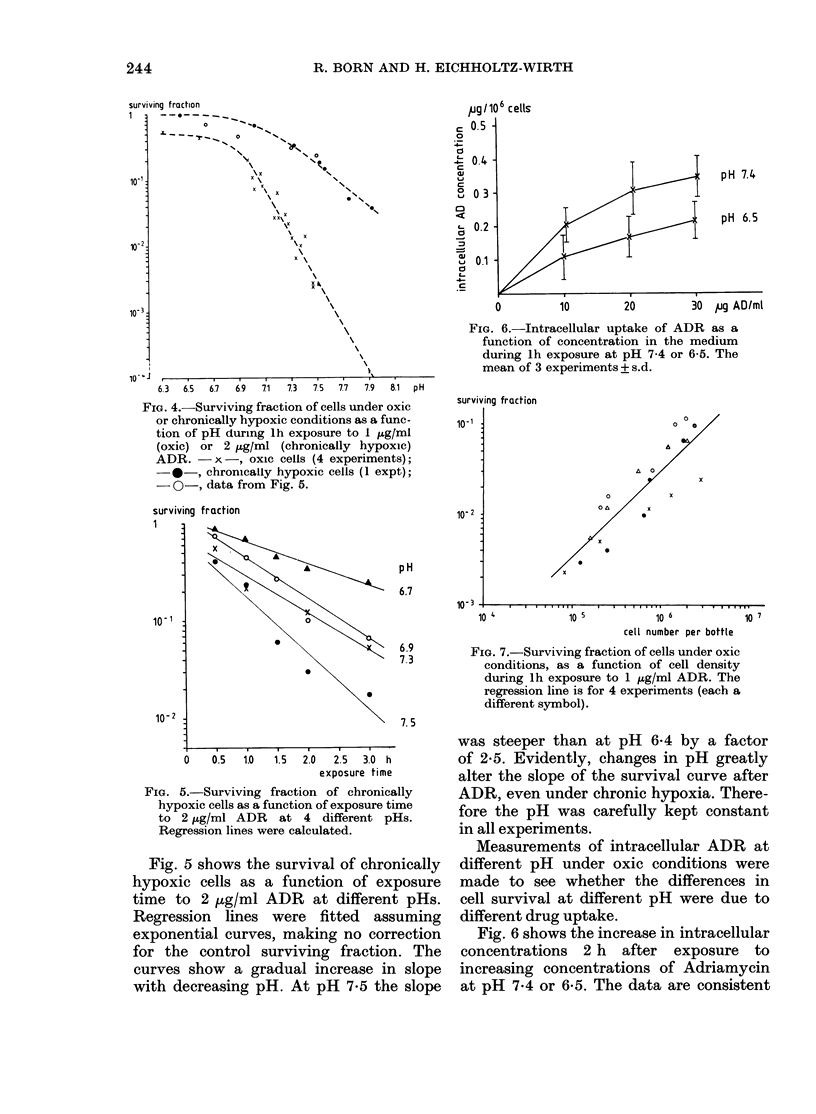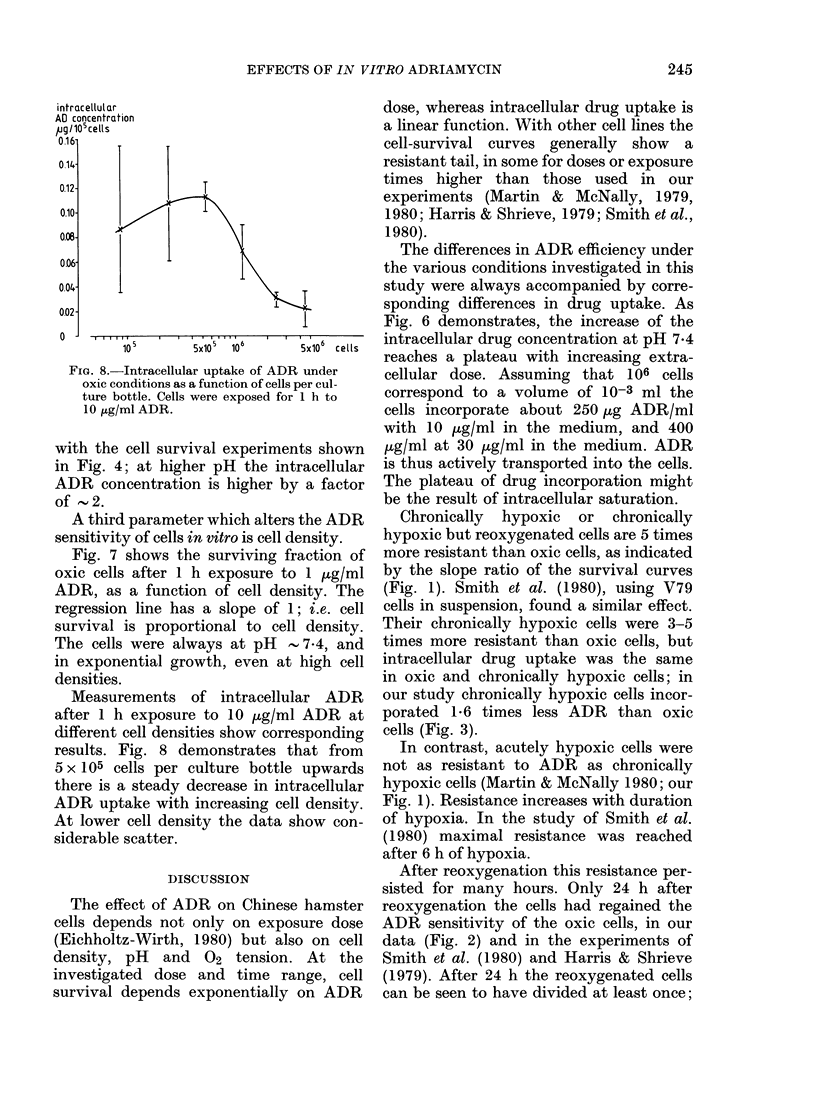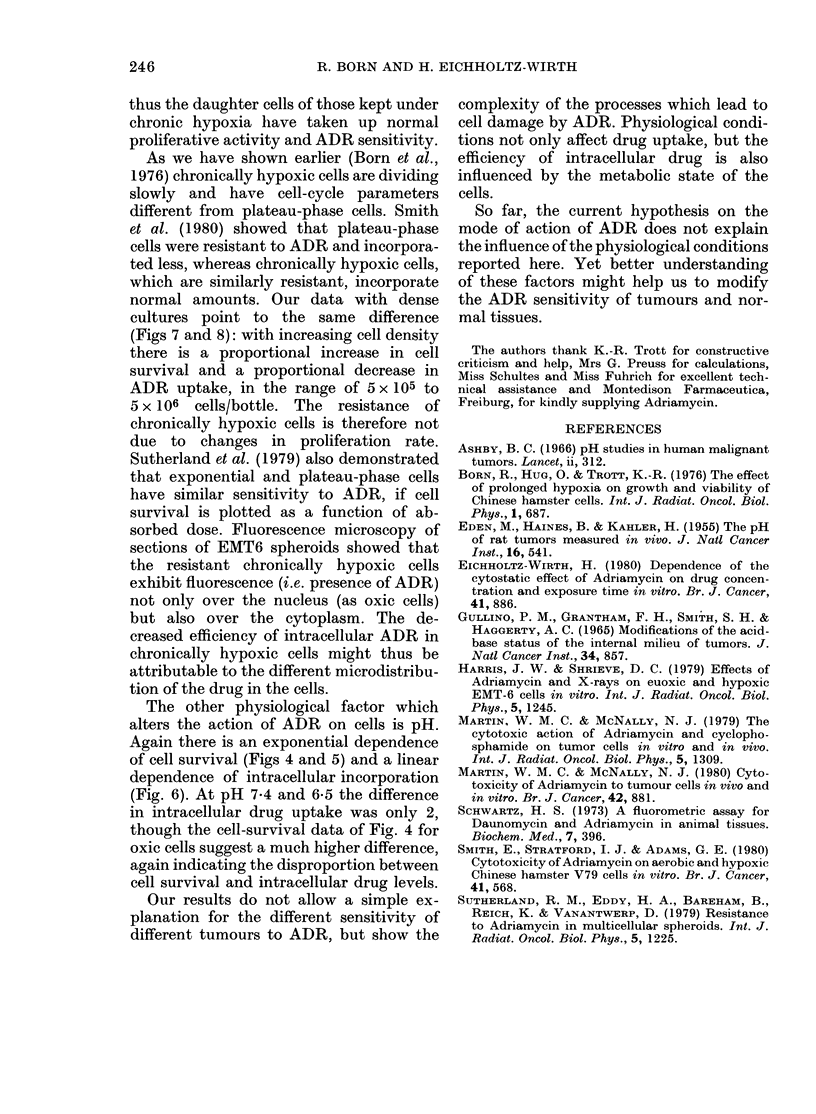# Effect of different physiological conditions on the action of adriamycin on Chinese hamster cells in vitro.

**DOI:** 10.1038/bjc.1981.175

**Published:** 1981-08

**Authors:** R. Born, H. Eichholtz-Wirth

## Abstract

Chronically hypoxic cells were 5 times more resistant to Adriamycin (ADR) than exponentially growing oxic cells. On reoxygenation, resistance decreased slowly to reach the ADR sensitivity of oxic cells after 24 h. With increasing pH, ADR efficiency increased more in oxic than in chronically hypoxic cells. With increasing cell density, ADR efficiency decreased linearly. The differences in ADR efficiency under the various conditions were accompanied by differences in intracellular ADR uptake. Chronically hypoxic cells incorporated 1.6 times less than oxic cells; the incorporation rate at pH 6.5 was half that at pH 7.4; and at a cell density of 5 X 10(5)/bottle the intracellular uptake was 6 times that at 5 X 10(6)/bottle. The observed differences in uptake of ADR were not, however, sufficient to explain the differences in cytotoxicity.


*Br. J. Cancer* (1981) **44**, 241

### EFFECT OF DIFFERENT PHYSIOLOGICAL CONDITIONS ON THE ACTION OF ADRIAMYCIN ON CHINESE HAMSTER CELLS *IN VITRO*

R. BORN* AND H. EICHHOLTZ-WIRTH

*From the *Abteilung für Strahlenbiologie der GSF, Neuherberg, and the Strahlenbiologisches Institut der Universitat München, W. Germany*

Received 17 February 1981 Accepted 13 April 1981

**Summary.—Chronically hypoxic cells were 5 times more resistant to Adriamycin (ADR) than exponentially growing oxic cells. On reoxygenation, resistance decreased slowly to reach the ADR sensitivity of oxic cells after 24 h. With increasing pH, ADR efficiency increased more in oxic than in chronically hypoxic cells. With increasing cell density, ADR efficiency decreased linearly.**

**The differences in ADR efficiency under the various conditions were accompanied by differences in intracellular ADR uptake. Chronically hypoxic cells incorporated 1·6 times less than oxic cells; the incorporation rate at pH 6·5 was half that at pH 7·4; and at a cell density of $5 \times 10^5$/bottle the intracellular uptake was 6 times that at $5 \times 10^6$/bottle.**

**The observed differences in uptake of ADR were not, however, sufficient to explain the differences in cytotoxicity.**

ADRIAMYCIN (ADR) is a common drug used in the chemotherapy of a variety of malignant diseases. In clinical as well as experimental research it has been shown that there are considerable differences in the ADR sensitivity between different tumours. Martin & McNally (1979) showed that the ADR resistance of mouse tumours was not necessarily due to the resistance of tumour cells as such. Consequently, the specific physiological conditions to which cells are exposed in the solid tumour, *i.e.* the cellular microenvironment, might be important for the cytotoxic action of ADR. We therefore investigated the dependence of ADR effectiveness on 3 factors which are specific features in tumours and can be copied *in vitro, viz.* acute and chronic hypoxia, changes of pH and cell density. These factors create physiological conditions which differ between tumours and exponentially growing cell cultures *in vitro*, and might contribute to the ADR resistance in some tumours. We measured the influence of these factors on the cytotoxicity and on the uptake of ADR into the cells *in vitro*.

### MATERIAL AND METHODS

B14 FAF 28 Chinese hamster cells were used. The cells were grown in Pyrex glass bottles with Eagle's minimal essential medium (MEM) supplemented with 10% calf serum, 100 mg/l neomycin and 350 mg/l $NaHCO_3$, and kept in a humid $CO_2$ incubator at 37°C.

The experiments were started after a 48h period of growth under normal conditions. For maintaining prolonged hypoxia the culture bottles were then continuously flushed with a mixture of 97% $N_2$ and 3% $CO_2$, as described elsewhere (Born *et al.*, 1976). After 12–14 h of gassing, when the cells were chronically hypoxic, the drug was added. Three hours of gassing was used to produce acute hypoxia. By controlling the $CO_2$ content of the gas mixture the pH in the culture bottles could be maintained at about 7·2, even during prolonged hypoxia. In the pH experiments with chronically hypoxic cells, the pH of the medium was adjusted by changing the $CO_2$ concentration of the gas mixture. In the experiments with oxic cells, the pH was adjusted by adding dilute NaOH or HCl.

Before the cells were plated after ADR exposure, the pH of each culture bottle was estimated by rapid aspiration of a sample of medium into a pH meter.

ADR was diluted in Hanks's solution and added directly to the growth medium in the culture bottles. Cells were incubated for 0·5–3·0 h with ADR at 37°C. In the experiments with acute or chronically hypoxic cells, 0·5 ml of ADR solution was added by a syringe through a small silicone stopper during continuous gassing and distributed by thorough shaking of the bottles. After exposure to ADR the cells were washed once for 5 min with Hanks's solution, trypsinized, diluted and seeded. The flasks were incubated for 7 days at 37°C, the colonies stained with methylene blue and counted.

The intracellular concentration of ADR was measured fluorometrically by the method of Schwartz (1973) as described in Eichholtz-Wirth (1980). After incubating the cells with ADR, the monolayer was rinsed with Hanks's solution for 1 h at 37°C to wash out all unbound ADR. Then the cells were cooled to 4°C, scraped off, centrifuged and resuspended in 1 ml of Hanks' solution. The cell concentration was $\sim 10^6$ per culture bottle; $2 \times 10^6$ cells were used per sample. To the 1ml cell suspension 0·2 ml of $AgNO_3$ (33% w/v) was added and shaken vigorously. After further addition of 3 ml iso-amyl alcohol, the solution was again shaken and then centrifuged at 1,000 rev/min for 5 min, when 2 ml of the upper phase was transferred to a cuvette. The fluorescence intensity was measured at a wave length of 580 nm, using an activation wave length of 483 nm.

FIG. 1.—Surviving fraction of Chinese hamster cells under oxic (—×—), acutely hypoxic (—△—), chronically hypoxic (—●—) or reoxygenated (—○—) conditions as a function of exposure time to 2 μg/ml Adriamycin. Each point represents the mean of 5 replicates of one independent experiment. The lines drawn for cells under oxic or acutely hypoxic conditions were fitted by eye.

### RESULTS

Exponentially growing Chinese hamster cells under normal conditions were much more sensitive to ADR than acutely or chronically hypoxic cells. Fig. 1 shows the survival of the cells as a function of duration of exposure to 2μg/ml ADR. All curves are exponential. Under aerated conditions the $D_0$ was 0·18 h. When the cells were acutely hypoxic the $D_0$ was 0·35 h. However, there was no significant difference between the survival of chronically hypoxic cells in comparison to oxic cells pretreated for the same time with hypoxia. The $D_0$ taken from the common regression line of chronically hypoxic and reoxygenated cells is 0·95 h. Thus an OER of 5 could be calculated from the slopes. Reoxygenation was achieved by a rapid medium change, and the drug was applied immediately afterwards. The resistance of the reoxygenated cells to ADR persisted for hours, as shown in Fig. 2. Cells were exposed for 1 h to 2 μg/ml ADR at different times after reoxygenation. About 24 h after reoxygenation, when the surviving cell population had divided at least once, the ADR sensitivity of oxic cells was reached.

To investigate the reason for the different toxicities of ADR to oxic and chronically hypoxic (hypoxic as well as reoxygenated) cells, intracellular concentrations of the drug were measured under both conditions. Fig. 3 shows the increase

FIG. 2.—Surviving fraction of reoxygenated chronically hypoxic cells as a function of time after reoxygenation. Cells were exposed to 2 μg/ml ADR for 1 h. The dashed line represents the survival of oxic cells not made previously hypoxic after the same ADR dose.

(a)

(b)

FIG. 3.—Intracellular uptake of ADR under oxic (—○—) or chronically hypoxic (—●—) conditions as a function of exposure time to 10 μg/ml ADR. (a) Data of a single experiment. Regression lines were calculated. (b) The mean value of 5 experiments with regression lines and error bars.

of ADR concentration in $2 \times 10^6$ cells with time of exposure to 10 μg/ml. Fig. 3a represents data of a single experiment at a cell density of $1{\cdot}5 \times 10^6$ cells per bottle and a pH of 7·2. The ADR concentration increased linearly with time. For the oxic cells the slope is 0·45 μg/h and for chronically hypoxic cells 0·27 μg/h per $2 \times 10^6$ cells. The ratio of the slopes is 1·7. This indicates that only part of the OER of 5 which was found for cell survival is due to differences in the rate of incorporation.

In Fig. 3b, 5 experiments with different cell densities ($1{\cdot}5$–$3{\cdot}5 \times 10^6$ cells/bottle) and pH (7·2–7·5) are pooled. The difference in the incorporation rate between oxic and chronically hypoxic cells is smaller than in the experiment with constant conditions. The incorporation rate is $0{\cdot}25 \pm 0{\cdot}03$ μg/h for oxic cells and $0{\cdot}17 \pm 0{\cdot}03$ μg/h for chronically hypoxic cells. The slope ratio is 1·5.

pH is another physiological factor which influences ADR toxicity. Between different tumours and tumour regions considerable differences in pH have been reported (Eden *et al.*, 1955; Gullino *et al.*, 1965; Ashby, 1966). We therefore investigated the influence of pH on the action of ADR. Fig. 4 shows cell survival after exposure to 1 μg/ml ADR under oxic or to 2 μg/ml under chronically hypoxic conditions for 1 h at different pH. The response of oxic cells to ADR is more sensitive to pH changes than is the response of chronically hypoxic cells.

FIG. 4.—Surviving fraction of cells under oxic or chronically hypoxic conditions as a function of pH during 1h exposure to 1 $\mu$g/ml (oxic) or 2 $\mu$g/ml (chronically hypoxic) ADR. —×—, oxic cells (4 experiments); —●—, chronically hypoxic cells (1 expt); —○—, data from Fig. 5.

FIG. 5.—Surviving fraction of chronically hypoxic cells as a function of exposure time to 2 $\mu$g/ml ADR at 4 different pHs. Regression lines were calculated.

Fig. 5 shows the survival of chronically hypoxic cells as a function of exposure time to 2 $\mu$g/ml ADR at different pHs. Regression lines were fitted assuming exponential curves, making no correction for the control surviving fraction. The curves show a gradual increase in slope with decreasing pH. At pH 7·5 the slope

FIG. 6.—Intracellular uptake of ADR as a function of concentration in the medium during 1h exposure at pH 7·4 or 6·5. The mean of 3 experiments ± s.d.

FIG. 7.—Surviving fraction of cells under oxic conditions, as a function of cell density during 1h exposure to 1 $\mu$g/ml ADR. The regression line is for 4 experiments (each a different symbol).

was steeper than at pH 6·4 by a factor of 2·5. Evidently, changes in pH greatly alter the slope of the survival curve after ADR, even under chronic hypoxia. Therefore the pH was carefully kept constant in all experiments.

Measurements of intracellular ADR at different pH under oxic conditions were made to see whether the differences in cell survival at different pH were due to different drug uptake.

Fig. 6 shows the increase in intracellular concentrations 2 h after exposure to increasing concentrations of Adriamycin at pH 7·4 or 6·5. The data are consistent

FIG. 8.—Intracellular uptake of ADR under oxic conditions as a function of cells per culture bottle. Cells were exposed for 1 h to 10 μg/ml ADR.

with the cell survival experiments shown in Fig. 4; at higher pH the intracellular ADR concentration is higher by a factor of ~2.

A third parameter which alters the ADR sensitivity of cells *in vitro* is cell density.

Fig. 7 shows the surviving fraction of oxic cells after 1 h exposure to 1 μg/ml ADR, as a function of cell density. The regression line has a slope of 1; *i.e.* cell survival is proportional to cell density. The cells were always at pH ~7·4, and in exponential growth, even at high cell densities.

Measurements of intracellular ADR after 1 h exposure to 10 μg/ml ADR at different cell densities show corresponding results. Fig. 8 demonstrates that from $5 \times 10^5$ cells per culture bottle upwards there is a steady decrease in intracellular ADR uptake with increasing cell density. At lower cell density the data show considerable scatter.

### DISCUSSION

The effect of ADR on Chinese hamster cells depends not only on exposure dose (Eichholtz-Wirth, 1980) but also on cell density, pH and $O_2$ tension. At the investigated dose and time range, cell survival depends exponentially on ADR dose, whereas intracellular drug uptake is a linear function. With other cell lines the cell-survival curves generally show a resistant tail, in some for doses or exposure times higher than those used in our experiments (Martin & McNally, 1979, 1980; Harris & Shrieve, 1979; Smith *et al.*, 1980).

The differences in ADR efficiency under the various conditions investigated in this study were always accompanied by corresponding differences in drug uptake. As Fig. 6 demonstrates, the increase of the intracellular drug concentration at pH 7·4 reaches a plateau with increasing extracellular dose. Assuming that $10^6$ cells correspond to a volume of $10^{-3}$ ml the cells incorporate about 250 μg ADR/ml with 10 μg/ml in the medium, and 400 μg/ml at 30 μg/ml in the medium. ADR is thus actively transported into the cells. The plateau of drug incorporation might be the result of intracellular saturation.

Chronically hypoxic or chronically hypoxic but reoxygenated cells are 5 times more resistant than oxic cells, as indicated by the slope ratio of the survival curves (Fig. 1). Smith *et al.* (1980), using V79 cells in suspension, found a similar effect. Their chronically hypoxic cells were 3–5 times more resistant than oxic cells, but intracellular drug uptake was the same in oxic and chronically hypoxic cells; in our study chronically hypoxic cells incorporated 1·6 times less ADR than oxic cells (Fig. 3).

In contrast, acutely hypoxic cells were not as resistant to ADR as chronically hypoxic cells (Martin & McNally 1980; our Fig. 1). Resistance increases with duration of hypoxia. In the study of Smith *et al.* (1980) maximal resistance was reached after 6 h of hypoxia.

After reoxygenation this resistance persisted for many hours. Only 24 h after reoxygenation the cells had regained the ADR sensitivity of the oxic cells, in our data (Fig. 2) and in the experiments of Smith *et al.* (1980) and Harris & Shrieve (1979). After 24 h the reoxygenated cells can be seen to have divided at least once; thus the daughter cells of those kept under chronic hypoxia have taken up normal proliferative activity and ADR sensitivity.

As we have shown earlier (Born *et al.*, 1976) chronically hypoxic cells are dividing slowly and have cell-cycle parameters different from plateau-phase cells. Smith *et al.* (1980) showed that plateau-phase cells were resistant to ADR and incorporated less, whereas chronically hypoxic cells, which are similarly resistant, incorporate normal amounts. Our data with dense cultures point to the same difference (Figs 7 and 8): with increasing cell density there is a proportional increase in cell survival and a proportional decrease in ADR uptake, in the range of $5 \times 10^5$ to $5 \times 10^6$ cells/bottle. The resistance of chronically hypoxic cells is therefore not due to changes in proliferation rate. Sutherland *et al.* (1979) also demonstrated that exponential and plateau-phase cells have similar sensitivity to ADR, if cell survival is plotted as a function of absorbed dose. Fluorescence microscopy of sections of EMT6 spheroids showed that the resistant chronically hypoxic cells exhibit fluorescence (*i.e.* presence of ADR) not only over the nucleus (as oxic cells) but also over the cytoplasm. The decreased efficiency of intracellular ADR in chronically hypoxic cells might thus be attributable to the different microdistribution of the drug in the cells.

The other physiological factor which alters the action of ADR on cells is pH. Again there is an exponential dependence of cell survival (Figs 4 and 5) and a linear dependence of intracellular incorporation (Fig. 6). At pH 7·4 and 6·5 the difference in intracellular drug uptake was only 2, though the cell-survival data of Fig. 4 for oxic cells suggest a much higher difference, again indicating the disproportion between cell survival and intracellular drug levels.

Our results do not allow a simple explanation for the different sensitivity of different tumours to ADR, but show the complexity of the processes which lead to cell damage by ADR. Physiological conditions not only affect drug uptake, but the efficiency of intracellular drug is also influenced by the metabolic state of the cells.

So far, the current hypothesis on the mode of action of ADR does not explain the influence of the physiological conditions reported here. Yet better understanding of these factors might help us to modify the ADR sensitivity of tumours and normal tissues.

The authors thank K.-R. Trott for constructive criticism and help, Mrs G. Preuss for calculations, Miss Schultes and Miss Fuhrich for excellent technical assistance and Montedison Farmaceutica, Freiburg, for kindly supplying Adriamycin.

### REFERENCES

ASHBY, B. C. (1966) pH studies in human malignant tumors. *Lancet*, ii, 312.

BORN, R., HUG, O. & TROTT, K.-R. (1976) The effect of prolonged hypoxia on growth and viability of Chinese hamster cells. *Int. J. Radiat. Oncol. Biol. Phys.*, **1**, 687.

EDEN, M., HAINES, B. & KAHLER, H. (1955) The pH of rat tumors measured *in vivo*. *J. Natl Cancer Inst.*, **16**, 541.

EICHHOLTZ-WIRTH, H. (1980) Dependence of the cytostatic effect of Adriamycin on drug concentration and exposure time *in vitro*. *Br. J. Cancer*, **41**, 886.

GULLINO, P. M., GRANTHAM, F. H., SMITH, S. H. & HAGGERTY, A. C. (1965) Modifications of the acid-base status of the internal milieu of tumors. *J. Natl Cancer Inst.*, **34**, 857.

HARRIS, J. W. & SHRIEVE, D. C. (1979) Effects of Adriamycin and X-rays on euoxic and hypoxic EMT-6 cells *in vitro*. *Int. J. Radiat. Oncol. Biol. Phys.*, **5**, 1245.

MARTIN, W. M. C. & McNALLY, N. J. (1979) The cytotoxic action of Adriamycin and cyclophosphamide on tumor cells *in vitro* and *in vivo*. *Int. J. Radiat. Oncol. Biol. Phys.*, **5**, 1309.

MARTIN, W. M. C. & McNALLY, N. J. (1980) Cytotoxicity of Adriamycin to tumour cells *in vivo* and *in vitro*. *Br. J. Cancer*, **42**, 881.

SCHWARTZ, H. S. (1973) A fluorometric assay for Daunomycin and Adriamycin in animal tissues. *Biochem. Med.*, **7**, 396.

SMITH, E., STRATFORD, I. J. & ADAMS, G. E. (1980) Cytotoxicity of Adriamycin on aerobic and hypoxic Chinese hamster V79 cells *in vitro*. *Br. J. Cancer*, **41**, 568.

SUTHERLAND, R. M., EDDY, H. A., BAREHAM, B., REICH, K. & VANANTWERP, D. (1979) Resistance to Adriamycin in multicellular spheroids. *Int. J. Radiat. Oncol. Biol. Phys.*, **5**, 1225.